# Fast Track Protocols and Early Rehabilitation after Surgery in Total Hip Arthroplasty: A Narrative Review

**DOI:** 10.3390/clinpract13030052

**Published:** 2023-04-25

**Authors:** Alberto Di Martino, Matteo Brunello, Davide Pederiva, Francesco Schilardi, Valentino Rossomando, Piergiorgio Cataldi, Claudio D’Agostino, Rossana Genco, Cesare Faldini

**Affiliations:** 1 1st Orthopedic and Traumatology Clinic, IRCCS Rizzoli Orthopedic Institute, Via G.C. Pupilli 1, 40136 Bologna, Italy; 2 Department of Biomedical and Neuromotor Science-DIBINEM, University of Bologna, 40136 Bologna, Italy

**Keywords:** enhanced recovery after surgery, fast track, total hip arthroplasty, peri-operative, post-operative, pre-operative

## Abstract

The Enhanced Recovery After Surgery (ERAS) or Fast Track is defined as a multi-disciplinary, peri- and post-operative approach finalized to reduce surgical stress and simplify post-operative recovery. It has been introduced more than 20 years ago by Khelet to improve outcomes in general surgery. Fast Track is adapted to the patient’s condition and improves traditional rehabilitation methods using evidence-based practices. Fast Track programs have been introduced into total hip arthroplasty (THA) surgery, with a reduction in post-operative length of stay, shorter convalescence, and rapid functional recovery without increased morbidity and mortality. We have divided Fast Track into three cores: pre-, intra-, and post-operative. For the first, we analyzed the standards of patient selection, for the second the anesthesiologic and intraoperative protocols, for the third the possible complications and the appropriate postoperative management. This narrative review aims to present the current status of THA Fast Track surgery research, implementation, and perspectives for further improvements. By implementing the ERAS protocol in the THA setting, an increase in patient satisfaction can be obtained while retaining safety and improving clinical outcomes.

## 1. Introduction

Enhanced Recovery After Surgery (ERAS) or Fast Track, is defined as the multi-disciplinary, peri- and post-operative approach performed to optimize patient’s health condition, reduce surgical stress, and improve post-operative recovery. It was introduced more than 20 years ago by Khelet et al. [1] to improve outcomes in patients undergoing general surgery and, in recent years, it has gained popularity also in orthopedic procedures [2].

ERAS protocols have been developed to promote a shorter convalescence and a faster functional recovery without an increase in the rates of morbidity and mortality; it implies the promotion of a reduced post-operative hospital length of stay (LOS) [3,4]. An appropriate Fast Track protocol needs to be tailored to the single patient and, through improved rehabilitation, it can significantly reduce pain and postoperative stress response, promoting a quicker psychological recovery [5]. To enable these protocols and obtain the best outcomes, it is necessary to build up a dedicated multidisciplinary team, including anesthesiologists, orthopedic surgeons, nurses, physical therapists, and sometimes even psychologists that adhere to specifically designed protocols on peri and postoperative care [6].

Total hip arthroplasty (THA) places a heavy financial load on the healthcare system, and it is, therefore, essential to optimize the available resources to speed up recovery and cut down the length of stay (LOS) without sacrificing outcomes [7]. Fast Track protocols allow a reduction in the need for hospitals’ bed capacity and related costs while improving patient experience and satisfaction [8], clinical safety, and clinical effectiveness [8,9,10,11,12,13]. Many hospitals have already successfully implemented protocols that allow the reduction of LOS without compromising outcomes [13], as confirmed by currently available literature that demonstrates how Fast Track programs in THA do not bear an increased risk of revision procedures or an increased risk of postoperative complications, including hip dislocations, postoperative re-admissions, and mortality [14,15,16,17,18]. However, as more and more studies prove the efficacy of Fast Track protocols, it has become clear that most surgeons focus on surgical technique alone, not considering the overall perioperative management of patients [19].

ERAS protocols should be based on the principle of “first better, then faster” [20]. The aim of minimizing LOS should not be obtained at the cost of worse outcomes [21] and several challenges remain to be fully addressed in terms of most suitable patients selection and management: reduction of subacute and persistent pain; optimizing opioid-sparing analgesic strategy; reduction of postoperative impairment of physical activity and function; further reduction of postoperative cognitive dysfunction; identification of high-risk patients for complications; anemia and transfusion thresholds; postoperative urine retention and urinary bladder catheterization; type, timing, and duration of physiotherapy [22].

**Figure 1:**
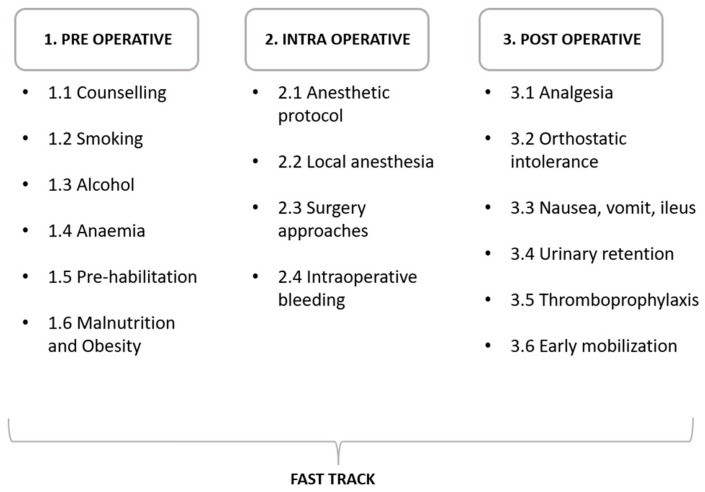
Fast Track protocols’ main steps divided according to the phase in preoperative, intraoperative, and postoperative.

The last review on the subject was done by Wainwright et al. [23] over 3 years ago, focusing on both hip and knee replacement. Since then, the literature on the subject has been expanded. This narrative review aims to implement the previous work by presenting the current status of clinical research in THA procedures performed using Fast Track protocols. For narrative purposes, the following review separately addresses three principal cores: pre-, intra-, and post-operative issues (Figure 1).

## 2. Pre-Operative Optimization

The first key step in THA surgery performed according to Fast Track protocols is patient selection. Traditionally, patients referred to a Fast Track protocol were young and fit. However, these limitations have been progressively overcome because of the implementation of minimally invasive surgical techniques and optimized anesthesiologic procedures; therefore, more patients are nowadays considered suitable for ERAS protocols in THA [24]. Ultimately, the objective is to select a patient that is functionally healthy pre-operatively, regardless of sex and age, that can withstand the surgery that is planned with minimal biological stress [24]. Overall, the standard inclusion criteria for THA surgery include primary surgery, ASA I-II, BMI <35, no pulmonary or cardiac functional limitation, previous normal hematocrit, no history of pulmonary embolism or deep vein thrombosis within the last six months, a limited requirement for urinary catheter positioning, preoperative normal cognitive function, and adequate home support [25,26]. The patient’s age has become a secondary factor for inclusion; rather than the number of years, the patient’s functional independence is recognized to be more relevant [25]. It is, therefore, recommended to evaluate the patient’s health globally and not only by age and BMI [26].

### 2.1. Counselling

Patient motivation and awareness are key to the implementation of ERAS protocols: not all patients may feel safe in being discharged early from the hospital. The psychological health of the patient influences physical recovery following THA surgery [27]; therefore, Fast Track protocols aim at achieving a high level of patient awareness through the improvement of preoperative education and information. The knowledge of general and critical aspects related to the surgery allows a decrease in anxiety [28,29,30,31], a well-known mental condition that affects cognitive functions, and promotes a more proactive rehabilitation [30]. Fast Track treatment also focuses on providing improved nutritional support, enforcing physical activity, and offering updated postoperative nursing care and rehabilitation [32,33,34]. The overall ERAS program works if patients have been thoroughly informed, and if they are encouraged to actively participate in their care and rehabilitation [29,35,36].

An additional key element that must be discussed with the patient in preoperative counseling is the presence of discharge criteria. These allow standardization of treatment goals and ensure that patients are discharged only when in a safe condition. Discharge criteria should be the following: stable ambulatory autonomy with crutch support and without dizziness; the absence of nausea or vomiting or a minimal presence that can be controlled without pharmacological intervention; pain <3 (according to the VAS scale) at rest and <5 during ambulation; and bleeding compatible with a normal postoperative course that does not require repeated dressing changes [37,38].

### 2.2. Smoking

Smoking is a known risk factor associated with a high rate of perioperative and postoperative complications, like myocardial infarction, cardiac arrest, pneumonia, urinary tract infection, sepsis, acute renal failure, and mortality [37,38,39]. These complications result in a longer hospital stay and higher total hospital costs [39]. It is, therefore, useful to encourage a cessation of smoking, to reduce adverse outcomes [40] and postoperative morbidity, beginning 6–8 weeks before surgery [41,42,43,44]. Patients should also be informed that bone integration of prosthetic implants, bone healing, and fusion are inhibited by cigarette smoking [43].

### 2.3. Alcohol Consumption

Like smoking, alcohol use can negatively influence patient outcomes after surgery [44]. When abused, it bears increased postoperative morbidity, with infections and cardiopulmonary insufficiency being the most frequent complications [45]. Moreover, alcohol misuse is associated with longer bleeding times in the first postoperative week and with an increased hospital stay [46]; conversely, abstainers show better improvement in the WOMAC osteoarthritis index function and pain control after THA surgery [47,48].

### 2.4. Anemia

Pre-operative anemia in the orthopedic setting is extremely common, with a prevalence ranging from 15 to 40% [49]. Anemia is associated with higher rates of morbidity and mortality, and it is one of the variables that most influences in-hospital LOS [50]. Implementation of pre-operative anemia management strategies, including the optimization of iron supplementation, decreases the rates of transfusions and complications, and the associated morbidity [51,52,53,54,55]. Chronic anemic patients, therefore, are not considered good candidates for ERAS protocols and should be redirected toward standard paths for THA surgery [27,56].

### 2.5. Pre-Habilitation

Reduced LOS requires rapid rehabilitation to allow the patient to regain functional autonomy. Traditionally, physical therapy is performed in the postoperative setting, and the patient regains the ability to walk and climb stairs with the assistance of a physical therapist that teaches the basis of walking with crutches, postoperative exercises, and avoidance of movements promoting dislocation. Several studies have recently shown that learning how to use crutches before THA performance and preparing the body with appropriate exercises before surgery decreases the time to independence, reduces LOS, and improves patients’ confidence [57,58]. These so-called pre-habilitation protocols can improve early postoperative pain and function among patients undergoing total joint replacement [59].

### 2.6. Malnutrition/Obesity

Malnutrition is a risk factor for postoperative complications; it slows tissue repair, extends in-hospital LOS, determines longer recovery times, and is associated with increased mortality [4]. Indicators of malnutrition status include low albumin, total lymphocyte count, and transferrin levels; these predict a prolonged recovery time and hospital stay after joint arthroplasty and, if possible, should be corrected before the surgical procedure [60]. At the other end of the spectrum, obesity is a risk factor for postoperative ischemic stroke, acute myocardial infarction, and cardiovascular death [61] and, following THA, for an increase in perioperative morbidity and mortality [61]. In THA procedures, obesity is linked to a higher risk of re-admissions and reoperations [62,63,64]. However, a recent study showed that only morbidly obese patients, adjusted for pre-operative co-morbidity, were associated with longer LOS [61].

Diabetes is a common comorbidity among obese patients. A pre-operative work-up is of the utmost importance in trying to reduce HbA1c below the 8% threshold associated with a higher incidence of wound complications and infections [24].

## 3. Peri-Operative Optimization

### 3.1. Anesthetic Protocol

Optimization of the anesthetic protocol is a major issue in patient candidates for Fast Track THA surgery. It requires specific team skills and a careful preoperative workup to support the patients in all the phases of the in-hospital stay. General and spinal anesthesia are the most common form of anesthetic protocols performed in this setting [65]. Different studies support spinal anesthesia over general anesthesia because of the provided benefits, including a decreased 30-day mortality rate, and reduced blood loss and complications [66]. Spinal anesthesia is linked to a shorter LOS, despite its more common association with postoperative nausea [65,67]; most important, its use is associated with overall lower scores on the pain numeric rating scales [68], as it decreases postoperative pain and can be easily combined with peripheral blocks and local anesthetics to promote recovery in a multimodal strategy of perioperative pain management [69,70,71,72]. Spinal anesthesia is, therefore, recommended for THA performance in Fast Track protocols to decrease LOS and short-term complications compared to general anesthesia [71,72,73].

### 3.2. Block/Local Anesthesia

To improve postoperative pain after THA, multimodal-analgesia can be implemented and opioid therapy dosage can be reduced by performing peripheral regional nerve blocks (PNB) [74]. PNB is recommended in THA as it reduces pain, allows early mobilization, and reduces morphine consumption while decreasing the risk of pain-related complications [75,76,77,78]. Different techniques can be adopted: femoral nerve block, sciatic nerve block, single shot, or continuous infusion [74]. This technique is beneficial for patients who undergo THA because it provides sufficient postoperative analgesia, especially against immediate postoperative resting pain [79,80]. Its relation to LOS is, however, still debated, and therefore, its routine use should be evaluated singularly [75].

Two other techniques that can take place in pain control are suprainguinal fascia iliaca block and pericapsular nerve group block (PENG), with the latter resulting in better motor functions [81,82], performed with a 20 mL infection of adrenalized levobupivacaine 0.50%. At the end of the procedure, the suprainguinal fascia iliaca block or a peri-incision infiltration can be administered with a solution composed of 100 mL of ropivacaine 2%, 1 mL of ketorolac 30 mg/mL, and 0.5 mL of adrenaline 1 mg/mL [81].

Peri-capsular infiltration is still a debated topic as a recent RCT showed no effect of local infiltration analgesia after THA [82]. More evidence is needed to make a clear statement on the matter.

### 3.3. Surgical Approaches

THA can be performed by different surgical approaches. Despite the recent trend toward minimally invasive muscle-sparing approaches [83], there is still no concrete proof that a specific surgical approach decreases LOS [23]. Minimally invasive approaches, by avoiding the gluteus medium, allow faster rehabilitation and reduced blood loss [84], usually at the cost of a longer learning curve [84,85]. Several studies have shown that DAA allows for a reduction in blood loss and perioperative pain [86], and for a faster recovery [87,88,89,90,91], which contributes to a reduction in LOS [89]. Moreover, in selected and well-prepared patients, an ultra-Fast Track protocol with a same-day discharge is possible when DAA is used [92,93]. More studies on the matter will be needed to obtain conclusive evidence on the best surgical approach to enable patients to return to normal as quickly as possible.

### 3.4. Intraoperative Bleeding

Antifibrinolytic agents have gained popularity to reduce bleeding and allogenic blood transfusions and, among them, tranexamic acid (TXA) certainly is the most extensively used [94,95]. TXA is commonly included in all Fast Track protocols to reduce the risk of postoperative anemia and the need for allogeneic blood transfusions [94], to decrease post-operative hematoma volume and eventually to reduce the incidence of postoperative heterotopic ossifications in the long term [96]. TXA, when feasible, is used in both old and young patients [97,98,99]. The best way to administer it (intracapsular, intravenous, mono, or multi-dose) is debated [95,100,101]. Current protocols warrant its use right before incision (its half-life is 80 min) with a dose of 10 mg/kg administered intravenously in a single infusion [99].

## 4. Post-Operative Optimization

### 4.1. Analgesia

**Figure 2:**
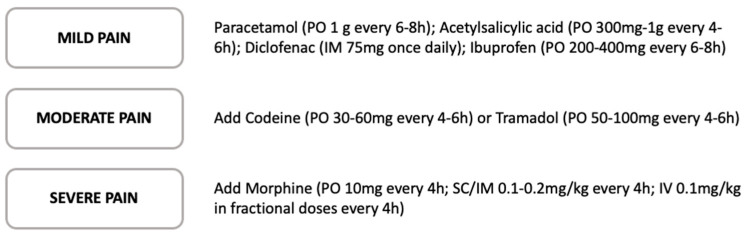
Step-analgesia protocol in relation to acute pain severity [107,108].

Multimodal analgesia for orthopedic surgery is now widely practiced as a means to reduce opioid use and opioid-related side effects. A multimodal approach is likely to produce superior analgesia than an opioid-only-based approach because multimodal analgesic agents target a variety of pain pathways [100]. Although all medications have side effects, opiates related complications are particularly concerning, showing multisystemic short and long-term side effects, including respiratory depression and nausea, that increase morbidity and prolong LOS after THA surgery [101]. For these reasons, multimodal analgesia aims to avoid or reduce opiate consumption by introducing other synergistic systemic agents, together with the use of regional or neuraxial blockade. Evidence supports regular doses of non-steroidal anti-inflammatory drugs (NSAIDs) in the postoperative period as an effective component of a multimodal, opioid-sparing regimen to manage acute pain; therefore, their use is recommended in all Fast Track guidelines [102]. Acetaminophen is well-tolerated and, when used in combination with NSAIDs or low-dosage opioids, it provides superior analgesia compared to single-agent NSAIDs or opiate regimes [103]. Gabapentin and pregabalin have both been demonstrated to reduce postoperative opiate requirements as part of a multimodal analgesia regimen [104] (Figure 2).

The use of a perioperative systemic glucocorticoid, although having robust evidence in knee replacement, is still debated in elective hip arthroplasty [105]. Their employment is neither associated with a higher complication rate nor a shorter hospital stay; however, they could find their role in selected patients [106]. More data on the subject is needed to provide a solid recommendation.

### 4.2. Orthostatic Intolerance

One of the problems associated with THA is the high incidence of an immediate postoperative orthostatic intolerance, which is defined as a drop >20 mmHg in systolic blood pressure or >10 mmHg in diastolic blood pressure associated with symptoms including dizziness, nausea, vomiting, vision disturbances, and syncope. This biological reaction, driven by an unknown impaired vasomotor response, has an incidence of 40% at 6 h and 20% at 24 h postoperatively [109], and it can cause falls and delays at the beginning of postoperative rehabilitation [110]. To date, orthostatic intolerance remains an unanswered therapeutic issue [111,112,113]. Therefore, a comprehensive preoperative work-up of the patient to adjust any antihypertensive therapy and the peri- and postoperative fluids administration aims to minimize the advent of this potentially serious condition [114,115,116,117,118].

### 4.3. Nausea, Vomit, Ileus

It is well known how these three complications slow the postoperative rehabilitation course; these are mostly attributable to the use of opioid drugs, and support the discontinuation or avoidance of opioid drugs in Fast Track multimodal analgesic protocols [115]. Of the three, ileus is the most feared complication since it can be associated with the need for a new hospitalization after discharge. Nausea and vomiting are known to delay hospital discharge only minimally, but newer anesthetic protocols that use multiple ways of administering pain medications have been implemented over the years to allow a reduction in the use and dosage of opioids [38,119,120].

### 4.4. Postoperative Urinary Retention

Postoperative urinary retention (POUR) is a common complication that occurs in more than 40 percent of patients subject to THA, especially when spinal anesthesia is performed. Although multiple pharmacological approaches are commonly used to prevent this event, when POUR occurs catheterization is required, either indwelling or intermittent [121,122]. Though not life-threatening, urinary retention and catheterization are associated with an increased risk of urinary infection, periprosthetic infection, and renal dysfunction [123,124]. All these invariably result in a slower rehabilitation course and a longer hospital stay [125]. One of the therapeutic options includes the reduction of opioid use through multimodal anesthesia, together with a good regulation of water homeostasis, as in many cases an excessive perioperative fluid administration can be detected [124,126,127,128,129,130]. Although urinary catheterization is not always avoidable, proper management of water balance and analgesic therapy can minimize this occurrence, the duration of which one should try to keep to a minimum [127].

### 4.5. Thromboprophylaxis

International guidelines on antithromboembolic prophylaxis are clear in suggesting extensive 10–30-day prophylaxis in high-risk procedures, including major orthopedic surgeries [131,132,133]. However, this concept has been questioned. Traditional guidelines were drawn up based on longer hospital stays during which mobilization was not performed as promptly and intensively as today. Recent studies set out to investigate whether shorter prophylaxis was as effective [130]. A study with more than 17,000 enrolled patients showed that in those with a hospital stay of 5 days or less, in-hospital-only administration of antithromboembolic prophylaxis was not associated with a higher risk of deep vein thrombosis or pulmonary embolism compared to traditional protocols [131]. The implementation of short-term antithromboembolic prophylaxis protocols, interrupted when the patient is fully ambulating, is a novel strategy that could further increase patient satisfaction and reduce the risk of postoperative bleeding while promoting wound healing [23,132].

### 4.6. Early Mobilization

Early function and mobilization are key factors for the success of a short-stay program, and recent Fast Track guidelines recommend that patients should be mobilized as soon as possible after surgery [23]. Mobilization on the day of surgery has gained importance in reducing LOS without increasing the rate of immediate adverse events, regardless of age, BMI, and ASA score [134,135,136,137,138,139,140]. Patients should be informed and educated about the postoperative physiotherapy progression: from isometric muscle contraction to assisted verticalization to ambulation with aids, and finally to climbing and descending stairs independently. As perioperative care continues to become more expedited, with more evidence suggesting the safety and patients’ satisfaction with outpatient THA [135,136], and health systems that aim to implement programs to speed recovery and reduce costs, it is important to identify barriers to early mobilization and find the means to overcome them.

## 5. Discussion

To maximize patient satisfaction and surgical outcomes while reducing costs and optimizing resources, Fast Track protocols were progressively introduced in THA surgery: a multi-disciplinary approach in the peri- and post-operative period, tailored to every patient (Figure 1).

Several studies have shown the advantages of the implementation of Fast Track protocols. Maempel et al. [137] studied over 1160 patients which performed THA, divided by rehabilitation protocol in ERAS or control group, and showed that patients treated by ERAS had a LOS shorter by an average of 1.5 days without an increased rate of postoperative complications. Carvalho et al. found [138] an even greater reduction in LOS (2.3 ± 0.8 days compared with 6.4 ± 1.5 days); however, in that study, most patients operated on by standard protocol required a higher blood transfusion and the use of a critical care unit, thus highlighting a difference in populations’ fragility.

Patients operated on according to ERAS protocols with early discharge from the hospital have long been considered at risk of subsequent re-admission, typically for surgical site infection, dislocation, deep vein thrombosis, or symptomatic anemia [139]. However, current evidence [140] shows that ERAS protocols are not relevant to re-admission rates. Sutton et al. [141] analyzed 19,000 THA divided by hospital discharge (within 2 days vs. 3 or 4 days following surgery), and found that early discharge was not an independent risk factor for 30-day major complications or re-admissions. Therefore, due to optimized patient selection, patients included in Fast Track protocols after THA are not at risk for increased re-admission rates compared to conventional pathways [110].

Patient selection was the critical issue in ERAS protocols, being more selective in the early years [142], but got progressively looser. It is now possible to adopt such protocols even in more vulnerable patients, including old fragile patients. Jørgensen et al. [143] investigated over 3000 patients who performed total hip or knee replacements using a Fast Track protocol. They found that more than 75% of those aged over 80 years had a LOS inferior to 4 days, with mortality and re-admission rate of 0.22% and 6.6%, respectively, at 30 days, and 0.42% and 9.3% at 90 days. Petersen et al. [26] prospectively studied a Fast Track protocol applied to a population over 85 years of 800 patients who performed THA. They found that it allowed a reduction of LOS and re-admission and mortality rate (11.7 and 0.9% after 30 days and 16.0 and 1.5% after 90 days, respectively).

Fast Track protocols are not without limitations. Patients need to be involved and well aware of the procedure. For this reason, patients with psychiatric conditions that may impede cognitive function and elderly patients with dementia are not eligible. Moreover, people with serious coagulation disorders or systemic diseases that may call for intensive care, numerous transfusions, or dialysis must be removed from the program. Another reason for exclusion could be the lack of nearby medical services or transportation issues since social and logistical support is of paramount importance in the period close to surgery to ensure a consonant rehabilitation pathway [24].

Fast Track practices can be implemented by an innovation introduced to cope with the COVID-19 pandemic. By reducing LOS, the use of Fast Track practices invariably results in reduced health professional-patient contact, just as during the outbreak. A more detached relationship is not associated with a reduction in clinical outcomes however, patients report a reduction in overall satisfaction [144]. An implementation of the protocol described in this paper would be the introduction of telemedicine in the postoperative follow-up. Indeed, telemedicine, by providing a form of professional–patient contact, is valuable both in maintaining high patient satisfaction and in providing patients with a rehabilitation pathway not inferior to that performed in person [145,146]. Future studies will be needed to confirm these hypotheses in accelerated inpatient regimens as well.

In conclusion, Fast Track THA surgery is effective in improving patient outcomes and satisfaction while decreasing healthcare costs and utilization of available resources. Optimization of the patient’s health preoperatively is crucial, together with appropriate patient selection in terms of general health status, motivation, commitment, and logistics. Pre-habilitation and customized post-operative rehabilitation protocols are also priorities. Future research will outline even more aspects to fine-tune ERAS protocols in THA candidate patients to minimize complications and costs while maximizing patient outcomes and satisfaction.

## Data Availability

No data available.
